# Primary Thrombophilia in Mexico XII: Miscarriages Are More Frequent in People with Sticky Platelet Syndrome

**DOI:** 10.4274/tjh.2016.0411

**Published:** 2017-08-02

**Authors:** Guillermo J. Ruiz-Delgado, Yahveth Cantero-Fortiz, Mariana A. Mendez-Huerta, Mónica Leon-Gonzalez, Ana K. Nuñez-Cortes, Andrés A. Leon-Peña, Juan Carlos Olivares-Gazca, Guillermo J. Ruiz-Argüelles

**Affiliations:** 1 Centro de Hematología y Medicina Interna de Puebla, Puebla, Mexico; 2 Universidad de las Américas Puebla, Puebla, Mexico; 3 Universidad Popular Autónoma del Estado de Puebla, Puebla, Mexico; 4 Laboratorios Clínicos de Puebla, Puebla, Mexico; 5 Benemérita Universidad Autónoma de Puebla, Puebla, Mexico

**Keywords:** Sticky platelet syndrome, thrombophilia, Miscarriages

## Abstract

**Objective:**

Sticky platelet syndrome (SPS) is an inherited condition that leads to arterial and venous thrombosis. There is scant information about the association between SPS and obstetric complications. This study aimed to assess the relationship between SPS and fetal loss at a single institution.

**Materials and Methods:**

The obstetric histories of all consecutive female patients prospectively studied in a 324-month period at a single institution with a history of thrombosis and a clinical marker of primary thrombophilia were reviewed.

**Results:**

Between 1989 and 2016, 268 consecutive patients with a clinical marker of primary thrombophilia and a history of arterial or venous thrombosis were studied; of these, 108 were female patients. Within this subset of thrombophilic females, 77 (71%) had been pregnant at some point. Twenty-eight of these 77 patients (37%) had had a spontaneous abortion and 24 of those (86%) were found to have SPS. On the other hand, in a subset of 73 female patients with SPS who had been pregnant, 32% had miscarriages. These figures are significantly higher than the prevalence of spontaneous abortions in the general Mexican population of pregnant women, which is 12%-13% (chi-square: 7.47; p=0.0063). Accordingly, the relative risk of having a miscarriage is 2.66 times higher in female patients with SPS than in the general population (p=0.0014).

**Conclusion:**

In Mexico, female patients with SPS experience significantly more spontaneous abortions than the general population. Since the treatment of SPS is simple and effective and could in turn prevent adverse obstetric outcomes, its investigation in women treated for obstetric complications may be useful and deserves further research.

## Abstract (tr)

**Amaç:**

Yapışkan trombosit sendromu (YTS), arteriyel ve venöz tromboza yol açan kalıtsal bir durumdur. YTS ve obstetrik komplikasyonlar arasındaki ilişki konusunda az bilgi vardır. Bu çalışma, tek bir merkezde YTS ve fetal kayıp arasındaki ilişkiyi değerlendirmeyi amaçlamıştır.

**Gereç ve Yöntemler:**

Tek bir merkezde, 324 aylık periyotta prospektif olarak incelenen, klinik belirteç olarak tromboz öyküsü ve primer trombofilisi olan kadın hastaların obstetrik hikayeleri gözden geçirilmiştir.

**Bulgular:**

1989 ile 2016 yılları arasında primer trombofili ve arteriyal veya venöz tromboz öyküsü olan 268 hasta çalışmaya alındı; bunlardan 108’i kadın hastalardı. Trombofilik bireylerin bir alt grubunda 77 (%71) kadın bir noktada hamile kaldı. Bu 77 hastanın 28’inde (%37) spontan düşük görüldü ve bunların 24’ünde (%86) YTS bulundu. Öte yandan, YTS’li 73 gebe kadın hastanın %32’lik bir kısmında düşük görüldü. Bu rakamlar, genel Meksika popülasyonundaki gebe kadınlardaki spontan düşüklerin prevalansından %12-%13 (ki-kare: 7,47; p=0,0063) daha yüksektir. Buna göre, düşük yapma göreceli riski YTS’li kadın hastalarda, genel nüfusa göre 2,66 kat daha fazladır (p=0,0014).

**Sonuç:**

Meksika’da YTS’li kadın hastalarda genel popülasyondan çok daha fazla spontan düşük görülmektedir.YTS’nin tedavisi basit ve etkilidir ve olumsuz obstetrik sonuçları önleyebilir. Bu nedenle obstetrik komplikasyonlar nedeniyle tedavi gören kadınlarda araştırılması faydalı olabilir ve daha fazla araştırma yapmayı hak eder.

## INTRODUCTION

Sticky platelet syndrome (SPS) was first described by Holliday et al. [1] at the 9^th^ Conference on Stroke and Cerebral Circulation in Arizona in 1983. Since then, we and others have found that SPS is a common cause of arterial and venous thrombosis [2,3,4,5,6,7,8,9,10,11,12,13,14,15,16,17]. SPS is the second most common hereditary thrombophilic condition after resistance to activated protein C and the most common thrombophilia associated with arterial thrombosis, with an incidence of 21% [3,4,5]. In Mexico, we have found that SPS is the second most frequent cause of hereditary thrombophilia, only surpassed by the 677 mutation in the *MTHFR* gene [4,5,17].

SPS is also the second most common thrombophilic condition that causes recurrent spontaneous abortions or fetal loss syndrome [18,19,20]. The diagnosis and classification of SPS relies on platelet aggregometry tests employing appropriate dilutions of two platelet aggregation inducers: adenosine diphosphate (ADP) and epinephrine [1,2,3,4,5,6]. In this study, we have assessed the relationship between SPS in female patients and their obstetric histories, focusing on the history of miscarriages.

## MATERIALS AND METHODS

### Patients

During a 324-month period, all consecutive mestizo Mexican patients referred to our center by physicians from different parts of the country were prospectively enrolled if they had one of the following clinical markers related to a primary hypercoagulable state [6,7,8,9,10,11]: a) thrombosis at age younger than 40 years; b) family history of thrombosis (first-degree relatives); c) recurrent thrombosis without apparent triggering factors; d) thrombosis at uncommon anatomic locations; or e) resistance to conventional antithrombotic therapy. Patients with overt malignancy, puerperium, pregnancy, use of oral contraceptives, or other conditions related to secondary thrombophilia were excluded. All patients had experienced at least one event of venous or arterial thrombosis as confirmed by either phlebography or Doppler; no surgery or trauma patients were included. The operational definition of “mestizo” given by Pons-Estel et al. [21] was employed to select patients as individuals born in Latin America who had both Amerindian and white ancestors. Informed consent was received from all the patients included in the study; the research was authorized by the Ethics and Human Research Subject Protection Committees of Clinica RUIZ. Spontaneous abortion or miscarriage was defined as a previous clinically recognized pregnancy loss as stated in the patient’s medical records.

### Analytical Methods

The following tests were done at least 3 months after the vaso-occlusive episode and patients were tested without the effect of anticoagulants or antiplatelets for at least 12 h.

For the assessment of SPS, we employed the procedure described by Mammen [4]: blood samples were drawn between 8:30 and 10:30 am via clean venipuncture with 19- or 21-gauge needles. After the venipuncture, the tourniquet was released and the first 5 mL was discarded. Then 18 mL of blood was aspirated into a 20-mL syringe containing 2 mL of 3.8% sodium citrate solution. The anticoagulated blood was centrifuged immediately for 10 min at 100 x g at room temperature to obtain platelet-rich plasma (PRP). After this, approximately one-half of the PRP was centrifuged a second time at 2000 x g for 20 min at room temperature to obtain platelet-poor plasma (PPP). In order to assess aggregation, the PRP was diluted with the PPP to achieve a platelet count of 200x10^9^/L. Platelet aggregation function was evaluated with an aggregometer (Model 500 CA, ChronoLog Corporation, Havertown, PA, USA), employing the original technique as described by Born and Cross [22]. Changes in optical density were recorded on a ChronoLog recorder (Model 703). While keeping the temperature (37 °C) and stirrer speed constant, aggregation was induced by exposure to three concentrations of ADP (2.34, 1.17, and 0.58 µM) and three concentrations of epinephrine (11, 1.1, and 0.55 µM) (final concentration in the PRP cuvette). We defined maximal aggregation as 100% light transmission, calibrated for each specimen. For each case normal controls were also studied. The cutoff points to define abnormal response for platelet aggregation with ADP exposure at 2.34, 1.17, and 0.58 µM were established as 55%, 36%, and 12%, respectively. The cutoff points to define abnormal response for platelet aggregation with epinephrine exposure at 11, 1.1, and 0.55 µM were established as 80%, 27%, and 20%, respectively. The presence of at least two abnormally high measurements out of the total six led to the diagnosis of SPS.

The activated protein C resistance (aPCR) phenotype was assessed using the ProC Global test kit (Siemens Healthcare Diagnostic Products GmbH, Marburg, Germany): aPCR was determined by the quantification of the increase in activated partial thromboplastin time in response to activated protein C using factor V-deficient plasma [8].

Coagulation protein C, coagulation protein S, antithrombin III, plasminogen, tissue-type plasminogen activator activity, plasminogen activator inhibitor activity, plasminogen activator inhibitor type, immunoglobulin G (IgG) and IgM isotypes of antiphospholipid antibodies, and lupus anticoagulants were also tested as was previously described [8].

For factor V gene mutations, a polymerase chain reaction (PCR)-based analysis for the factor V p.506R>Q (Leiden) mutation was performed according to the method of Zöller and Dahlbäck [23]. A region of the factor V gene from nucleotide 1690 to nucleotide 1692 of codon 506 was amplified by PCR and then subjected to digestion with MnII restriction endonuclease. The restriction pattern was studied by electrophoresis on a 4.5% polyacrylamide gel. The used oligonucleotides were those described by Bick [3]. To investigate the factor V HR2 haplotype, exon 13 was amplified with primer pair F5 13-1 and F5 13-2 [24]. The polymorphism p.1299H>R, which forms a part of allele R2, was then observed by restriction analysis with RsaI [9]. For detection of the p.306R>T (Cambridge) mutation, exon 7 of the factor V gene was amplified using primers F5 7-1 and F5 7-2 [25]. The mutation was determined by restriction analysis with BstNI. For the factor V gene Hong Kong mutation (p.306R>G), the 241-bp amplification product achieved for detection of the factor V Cambridge mutation was digested with HpaII according to Chan et al. [26]. For the factor V gene Liverpool mutation (p.359I>T), exon 8 of the factor V gene was amplified using primers F5 8-1 and F5 8-2 [9]. The amplification product achieved for the identification of the factor V Liverpool mutation was digested with BsrI according to Mumford et al. [27].

Identification of transition c.677C>T of the 5,10-methylenetetrahydrofolate-reductase (*MTHFR*) gene was performed based on the procedure originally described by Kluijtmans et al. [28]. A fragment of the *MTHFR* gene from nucleotide 598 to nucleotide 705 was amplified. To establish the genotype, the amplicon was treated with HinfI. Homocysteine levels were not assessed.

A-PCR-based analysis for the c.20210G>A polymorphism in the 3’-untranslated region of the prothrombin gene was performed as described by Poort et al. [29]. By PCR, a section of the prothrombin gene from nucleotide 19889 to nucleotide 20212 was amplified. In this process, a mutation is introduced by one of the primers that generates a HindIII restriction site along with the G>A mutation. An aliquot of the amplified product was therefore digested with HindIII restriction endonuclease.

### Statistical Analysis

Statistical analyses were performed using the chi-square test while comparing categorical variables. For all analyses, p<0.05 was used to indicate statistical significance.

## RESULTS

A total of 268 consecutive Mexican mestizo patients were prospectively enrolled in this study following their referral to our clinic by physicians from various parts of the country based upon the criteria described above. In addition to having a clinical marker of primary thrombophilia as previously explained, all of them had suffered at least one event of venous thrombosis as confirmed by either phlebography or Doppler, and they were not receiving antiplatelet drugs in the previous 12 h; accordingly, all of these individuals were patients with primary and/or secondary thrombophilic conditions [6,17]. From this group, a subset of 108 thrombophilic female patients was selected for further analysis; of these, 77 (71%) had been pregnant at some time, and within that subset, 28 (37%) had experienced at least one spontaneous abortion. Within the group of patients who had experienced spontaneous abortion, the prevalence of SPS was 86% (24 out of 28); the remaining four patients who had experienced abortion were found to be heterozygous for the 677C>T mutation of the *MTHFR* gene. In the subset of female patients who had been pregnant and had no abortions, the prevalence of SPS was 63% (31 out of 49) and the prevalence of the 677C>T mutation of the *MTHFR* gene was 37% (18 of 49). It is clear that the prevalence of SPS in patients who had been pregnant and had had a miscarriage is higher (86% versus 63%) and it is also higher than the prevalence of SPS in the general population (86% versus 15%).

**Figure 1:**
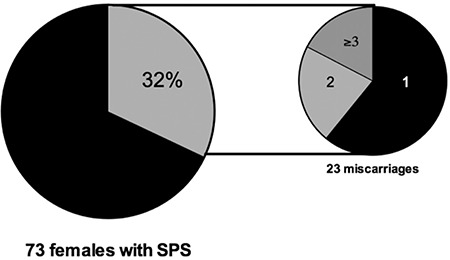
Twenty-three of 73 (32%) female patients with sticky platelet syndrome experienced an abortion at some time. Fourteen patients had one abortion, 5 had two, and 4 had three or more. SPS: Sticky platelet syndrome.

On the other hand, in a subset of 73 female patients with SPS who had experienced a pregnancy, 23 (32%) had lost their pregnancies: 14 patients had had an abortion, 5 had had two abortions, and 4 had had three or more abortions. These data are significantly different from those observed in the general Mexican pregnant population, in which 12%-13% of pregnancies end in a spontaneous abortion [30] (chi-square: 7.47, p=0.006), and they indicate that the relative risk of having a miscarriage is 2.66 times higher in Mexican female patients with SPS than in the general population (p=0.001) (Figure 1). Other causes of fetal loss were not found in the files of the patients. Table 1 shows the outgoing data of the thrombophilic studies done in the 23 patients with SPS who had experienced at least one miscarriage. It is interesting that 9/23 displayed the *MTHFR* gene mutation and that 7 additionally had antiphospholipid antibodies, the prevalence of the *MTHFR* gene being lower in this subset of patients than that previously reported by us in both thrombophilic individuals and the general population.

## DISCUSSION

Pregnant women may experience a variety of adverse obstetric events, such as preeclampsia, placental abruption, fetal growth retardation, and pregnancy loss, and these may be related to alterations in placental perfusion. The low pressure and turbulent flow pattern of circulation at the placenta, along with changes in hypercoagulability during this period, may also predispose women to thrombosis [31]. While the association between venous or arterial thromboembolism and inherited thrombophilias is widely recognized, no definitive relationship between adverse pregnancy outcomes and inherited thrombophilias has been successfully proven to date. It is possible that some hypercoagulable states may predispose individuals to arterial thrombosis, which can then result in uteroplacental thrombosis and obstetric complications. In a retrospective review of 351 women evaluated for recurrent pregnancy loss, Bick and Hoppensteadt [18] identified SPS in 64 of them (18%). Other authors have also shown increased prevalence of obstetric complications in patients with SPS [18,19,31]. For example, our group demonstrated that, in the general Mexican population, the prevalence of SPS and the 677 mutation in the *MTHFR* gene is 15% and 79%, respectively [16,17].

In Mexico, 12%-13% of pregnancies in the general population end in a spontaneous abortion [31]; this figure is significantly lower than that which we have found in the group of patients with SPS (32%; chi-square: 7.47, p=0.006). In turn, the relative risk of having a miscarriage is 2.66 times higher in female patients with SPS than in the general population (p=0.001).

## CONCLUSION

Our study was not intended to address the prevalence of SPS in pregnant women. The figures presented here suggest that this inherited condition may be related to obstetric complications, and SPS accordingly may be developing as an etiology for adverse pregnancy outcomes. However, there is a shortage of literature on this syndrome in the pregnant population. Since the treatment of SPS is simple, cheap, and highly effective, employing aspirin and/or other antiplatelet drugs [13,16], the investigation of SPS in women treated for obstetric complications may be useful and deserves further research. The appropriate use of aspirin and/or other antiplatelet drugs during the pregnancy of patients with SPS could result in the prevention of adverse pregnancy outcomes.

## Figures and Tables

**Table 1:**
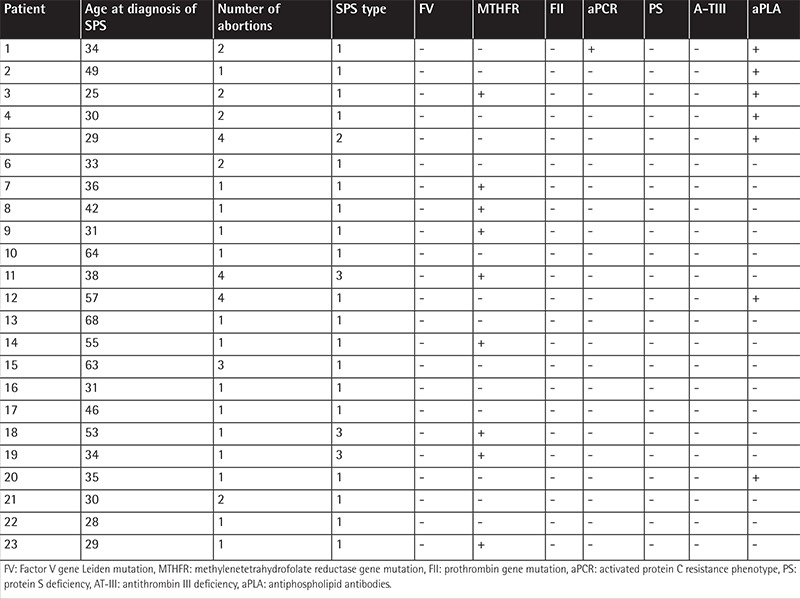
Salient features of the thrombophilia studies of the 23 female patients with sticky platelet syndrome who experienced an abortion.
